# Myths in magnetosensation

**DOI:** 10.1016/j.isci.2022.104454

**Published:** 2022-05-23

**Authors:** Simon Nimpf, David A. Keays

**Affiliations:** 1 Division of Neurobiology, Faculty of Biology, Ludwig-Maximilians-University Munich, Planegg-Martinsried, 82152 Munich, Germany; 2 University of Cambridge, Department of Physiology, Development & Neuroscience, Downing Street, CB2 3EG Cambridge, UK; 3 Research Institute of Molecular Pathology (IMP), Vienna Biocenter (VBC), Campus- Vienna-Biocenter 1, 1030 Vienna, Austria

**Keywords:** Biological science, ethology, zoology, physiology

## Abstract

The ability to detect magnetic fields is a sensory modality that is used by many animals to navigate. While first postulated in the 1800s, for decades, it was considered a biological myth. A series of elegant behavioral experiments in the 1960s and 1970s showed conclusively that the sense is real; however, the underlying mechanism(s) remained unresolved. Consequently, this has given rise to a series of beliefs that are critically analyzed in this manuscript. We address six assertions: (1) Magnetoreception does not exist; (2) It has to be magnetite; (3) Birds have a conserved six loci magnetic sense system in their upper beak; (4) It has to be cryptochrome; (5) MagR is a protein biocompass; and (6) The electromagnetic induction hypothesis is dead. In advancing counter-arguments for these beliefs, we hope to stimulate debate, new ideas, and the design of well-controlled experiments that can aid our understanding of this fascinating biological phenomenon.

## Graphical abstract

**Figure:**
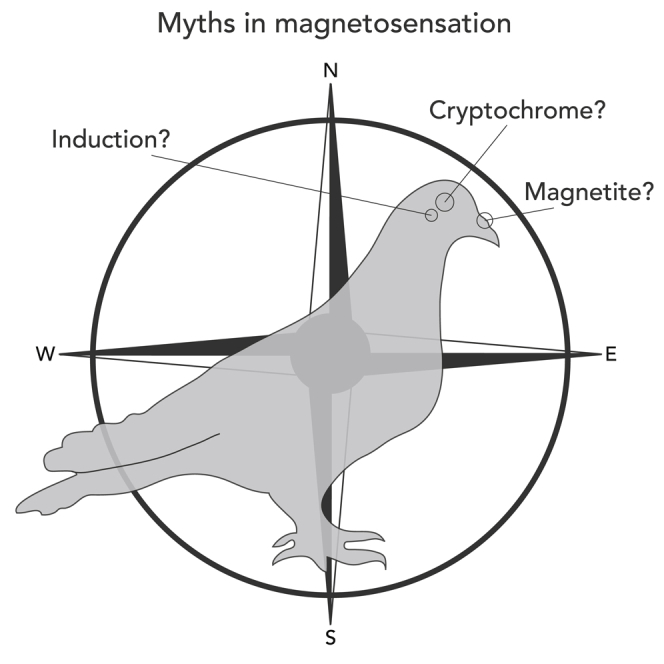

## Introduction - magnetoreception does not exist

“What could be the physical force everywhere present in the heights of the atmosphere as well as the depths of the oceans that could direct animals that migrate? In my opinion, there is only one…… and that is the Earth’s magnetic field” (Viguier, 1882).

**Figure 1:**
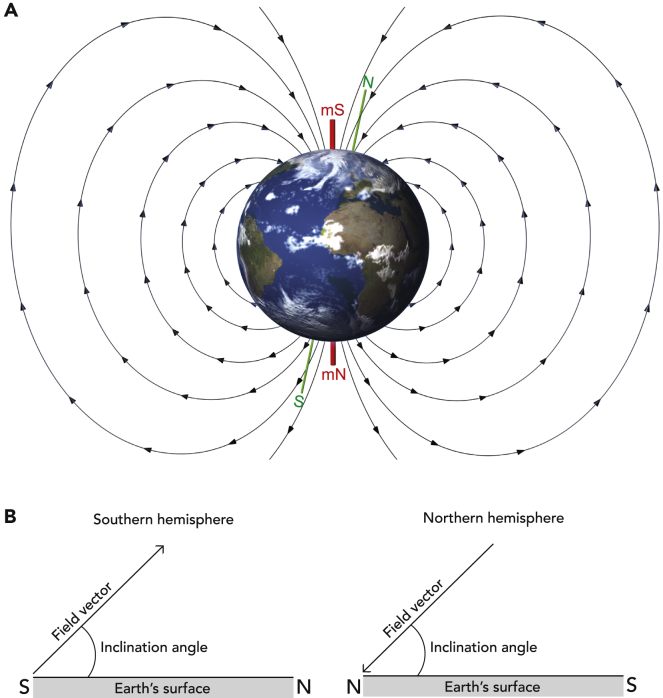
The Earth’s magnetic field and its components (A) The Earth is surrounded by a static magnetic field that is believed to result from movement of the iron core at its center. The magnetic field lines emanate from the magnetic North Pole (mN) in the southern hemisphere and enter the Earth at the magnetic South Pole (mS) in the northern hemisphere. The intensity of the magnetic field is proportional to the distance between the magnetic field lines. The intensity gradient on the planet ranges from ∼65 μT at the poles to ∼25 μT at the equator. (B) The inclination of the Earth’s magnetic field is defined as the angle between the magnetic field lines and the Earth’s surface, which changes from ∼90° at the poles to 0° at the magnetic equator.

With these words, published in 1882, Viguier was among the first to predict the existence of a magnetic sense; however, it was not an idea that was embraced by the scientists of his day. In 1898, Fritz Braun, a well-known ornithologist argued that “until we are in the possession of a magnetic or electric sense, these theories are comparable to an individual of a blind species trying to come up with a theory of light…. The assumption of a specific sense in migratory birds, which is essentially different from that in humans, is, for epistemological reasons unacceptable” (Braun, 1898). This objection, that we simply cannot understand a sense that we do not possess, might seem short sighted today but Fritz’s remarks highlight one of the primary difficulties that the field of magnetosensation has faced. The design of experiments that explore the mechanistic basis of hearing, for instance, is conceptually much simpler. An experimenter can detect the presence of artifacts, appreciate the texture of the stimulus, identify the likely anatomical location of the primary sensors, and instinctively imagine how this information is utilized by the organism. The same cannot be said for magnetic stimuli, particularly when we consider that the Earth’s magnetic field has three components: (1) a polarity, (2) an inclination, and (3) intensity (Figure 1). Each of these parameters might be detected by an animal, potentially by different sensory systems in different anatomical loci.

The breakthrough experiment came in 1965 when Wolfgang Wiltschko, a doctoral student in the group of Friedrich Merkel, published the first compelling experimental evidence for a magnetic sense in European robins (*Erithacus rubecula*). They focused on the bird’s migratory restlessness, a seasonally dependent movement that reflects the preferred orientation of the birds had they been freely flying. Merkel and Wiltschko showed that altering the magnetic environment leads to a deflection of the preferred migratory direction (Merkel and Wiltschko, 1965). Wolfgang and Roswitha Wiltschko built on this work, and in 1972 published a beautiful paper that showed that robins do not rely on the polarity of the magnetic vector for orientation, but rather the inclination of the field with respect to the surface of the Earth (Wiltschko and Wiltschko, 1972). Drawing on increasingly sophisticated behavioral tests and the use of double-wrapped coil systems to control for experimental artifacts, investigators have now convincingly demonstrated that many species of the planet have a magnetic sense (Putman, 2022; Wiltschko and Wiltschko, 2021; Beaugrand, 1977). The list of magnetosensitive species includes: bacteria, prokaryotes, honey bees, cockroaches, newts, turtles, rodents, lobsters, fish, bats, and both migratory and non-migratory birds (Freire et al., 2005; Boles and Lohmann, 2003; Holland et al., 2006; Phillips, 1986; Blakemore, 1975; Keim et al., 2004; Bazalova et al., 2016; Nemec et al., 2001; Naisbett-Jones and Lohmann, 2022). The important question is no longer: Do animals detect the Earth’s magnetic field? But rather: How do animals detect magnetic fields?

## It has to be magnetite

**Figure 2:**
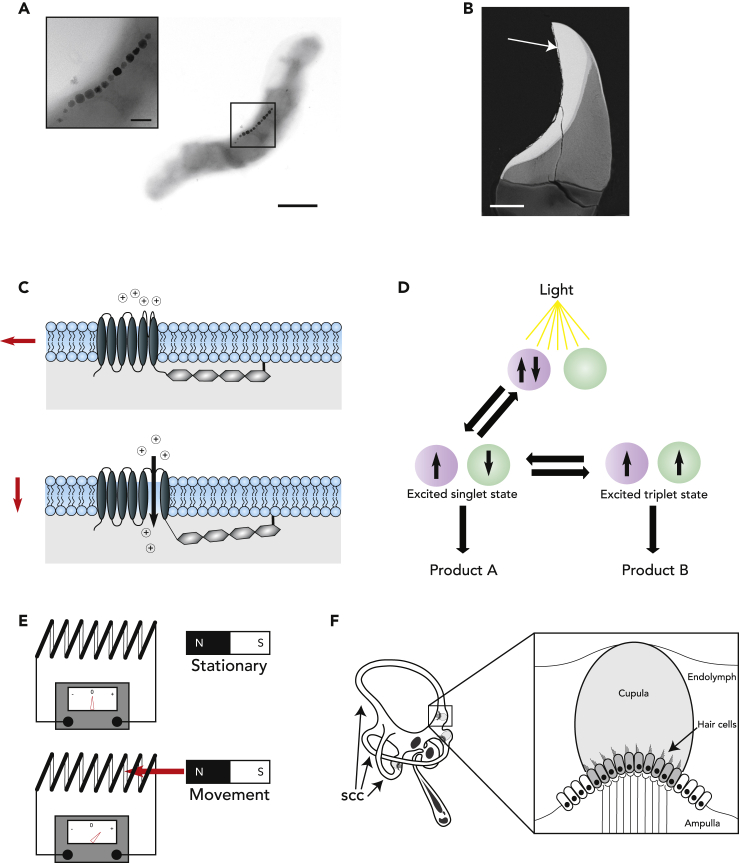
Proposed mechanisms of magnetoreception (A) Electron micrograph of *Magnetospirillum magnetotacticum* with a chain of magnetite crystals located inside the cell body. Magnetotactic bacteria are aquatic and anoxic. They employ intracellular magnetic crystals to limit three-dimensional swimming, favoring linear movement along the Earth’s magnetic vector. This enables them to more readily locate a depth with the optimal oxygen concentration (Uebe and Schuler, 2016). (B) Scanning electron microscope image of single major lateral tooth from the chiton *Acanthopleura hirtosa*. The magnetite mineralization zone is highlighted with a white arrow. (C) Schematic illustrating the magnetite-based hypothesis, whereby crystals of magnetite are tethered to a mechanosensitive cation channel. Depending on the orientation of the Earth’s magnetic vector (shown with red arrows), a torque is exerted on the channel thereby closing or opening it. (D) Schematic illustrating the light-induced radical pair formation hypothesis. Light causes an electron transfer between two molecules, resulting in a singlet state or a triplet state. The interconversion of these two states can be influenced by magnetic fields, thereby potentially altering the ratio of the products A and B. (E) Schematic illustrating the theory of electromagnetic induction. A changing magnetic field can induce a voltage in a conductive material, which can be measured by a galvanometer. (F) A schematic representation of the avian inner ear highlighting the semicircular canals (scc), that are filled with endolymph. The cristae ampullaris are located at the base of each semicircular canal, one of which is shown in an enlarged box. Each crista ampullaris contains sensory hair cells, the stereocilia of which are embedded in the cupula. It has been proposed that if birds rotate their head such that the plane of a semicircular canal changes with respect to the Earth’s magnetic vector, a voltage could by induced in the endolymph. Provided that the cupula prevents or limits ionic flow, the difference in electric potential could be detected by voltage-sensitive hair cells. The scale bar in panel (A) shows 500 nm and 100 nm in the inset. The scale bar in panel (B) shows 50 μm.

One of the dominant theories in the field of magnetosensation predicts that the iron oxide magnetite (Fe_3_O_4_) is the underlying substrate that enables the conversion of a magnetic stimulus into a neuronal impulse (Figure 2C). This idea emerged with the discovery of magnetotactic bacteria, which are characterized by their ability to align and swim along the Earth’s magnetic field lines to reach preferred environmental conditions (Lefevre et al., 2014; Blakemore, 1975). These bacteria contain specialized organelles, known as magnetosomes, that consist of multiple single-domain crystals of magnetite that collectively possess sufficient magnetic moment to act as an intracellular rudder (Figure 2A). With the discovery that chitons are able to generate bilateral rows of teeth made of this iron oxide, it was apparent that the ability to produce biogenic magnetite was not limited to bacteria (Figure 2B) (Lowenstam, 1962). This led a number of investigators to propose that magnetosensitive animals might employ an intracellular magnetite-based compass that is coupled to mechanically sensitive cation channels (Figure 2C) (Kirschvink et al., 2001; Lohmann and Johnsen, 2000; Walker, 2008; Cadiou and Mcnaughton, 2010). This concept has been supported by a series of behavioral experiments that involve subjecting an animal to an intense magnetic stimulus (between 0.1 and 5T) that would re-polarize a magnetoreceptor based on single domain magnetite. For instance, Holland and Helm exposed migrating European robins to a magnetic pulse and reported that this altered the departure direction of adult animals (but not juveniles) when they were released (Holland and Helm, 2013). It is important to appreciate, however, that these experiments are not proof of single domain magnetite. To address this, numerous labs have attempted to find biogenic magnetite in an array of species. Anatomical regions of interest have included the olfactory epithelium of trout (Walker et al., 1997; Eder et al., 2012), the abdomen of honeybees (Kuterbach et al., 1982), the upper beak skin of pigeons (Fleissner et al., 2003, 2007), and the antennae of ants (Abracado et al., 2005). In undertaking this search, investigators have used a variety of methods including the histological stain Prussian blue (PB) (Fleissner et al., 2003), magnetic screening of trypsinated cells (Eder et al., 2012), energy electron loss spectroscopy (EELS) (Edelman et al., 2015), and synchrotron X-ray-based methods (Fleissner et al., 2007; Malkemper et al., 2019). As magnetite crystals are very small (≈40nm), the search for them was initially compared to finding a needle in a haystack, but a better analogy that has been proposed is the search for a needle in a haystack of needles—and for good reason (Shaw et al., 2015; Johnsen and Lohmann, 2008). Iron is essential for life and is found in many cell types, predominantly as ferrihydrite nanocrystals within the ferritin protein supercomplex (Chasteen and Harrison, 1999). Moreover, as magnetic fields pass freely through biological tissue, the primary receptors could be located anywhere in the animal. The search has been further compromised by the ease of which biological samples are contaminated with magnetic particles. The laboratory serves as a fertile source of magnetic material whether it be from glassware, dissection tools, or histological blades (Edelman et al., 2015; Kobayashi et al., 1995). The magnetite theory therefore awaits a smoking gun, a transmission electron micrograph coupled with elemental analysis that clearly and reproducibly shows crystals of intracellular magnetite in sensory cells of a defined anatomical locus. Such data are required to transform this myth into fact.

## Birds have a conserved six loci magnetic sense system in their upper beak

**Figure 3:**
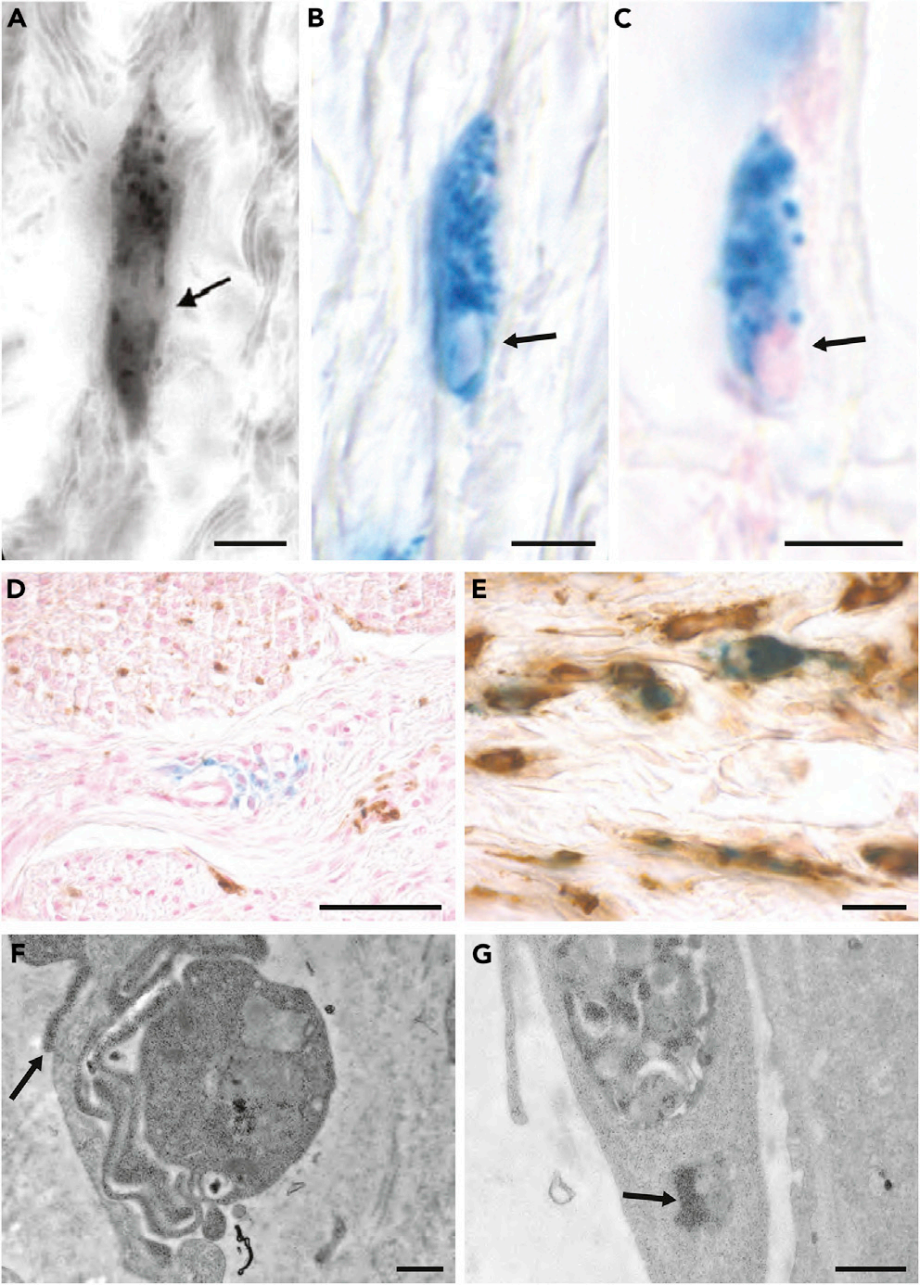
Iron-rich cells in the upper beak of pigeons are macrophages not magnetosensitive neurons (A) Image of a cell stained with Prussian blue (PB) from the upper beak skin of pigeons taken by Fleissner and colleagues (Falkenberg et al., 2010). They claimed that the image shows a dendritic projection of the ophthalmic branch of the trigeminal nerve, and that the punctate bullets and central vesicle (arrowed) are part of a magnetosensory apparatus. (B) Image of a PB-positive cell from the pigeon upper beak taken by the Keays laboratory shows a strikingly similar morphology with a light blue cytoplasmic stain, 20–30 blue “bullets” and a central vesicle (arrowed). (C) Staining with nuclear fast red and PB reveals that the central vesicle is a nucleus (arrowed). (D) Image of a histological section stained with PB and sera against the post mitotic neuronal marker TuJ (brown staining), revealing that PB-positive cells in the pigeon upper beak are not neurons. (E) Image showing that PB-positive cells co-localize with CD44 (brown staining), a cell surface marker that labels white blood cells, including macrophages. (F–G) Transmission electron micrographs of PB-positive cells from the pigeon upper beak. At a subcellular level, the cells are characterized by the presence of cytoplasmic ferritin (accounting for the background cytoplasmic stain), siderosomes in which ferritin is nucleated (arrowed in G), and the presence of filopodia (arrowed in F). Scale bars in A, B, and C show 5 μm. Scale bars in D and E show 10 μm. Scale bars in F and G show 1 μm.

The anatomical locus of the primary magnetosensors in birds has been hotly contested, particularly with respect to the upper beak of birds. Over the years, a number of papers have implicated the trigeminal system in magnetosensation (Mouritsen Hore, 2012). Mora and Walker have reported that severing the ophthalmic branch of the trigeminal nerve compromises magnetic conditioning in pigeons (Mora et al., 2004); Mouritsen and colleagues have shown that magnetic stimuli, in multiple bird species, increase the number of neurons expressing the immediate-early gene ZENK in the primary trigeminal (PrV) and spinal trigeminal (SpV) nuclei (Heyers et al., 2010; Lefeldt et al., 2014; Elbers et al., 2017); and a magnetic map sense in migratory reed Warblers has been associated with an intact trigeminal system (Kishkinev et al., 2013). As the ophthalmic branch of the trigeminal nerve innervates the upper beak and ocular regions (Wild et al., 1984), Fleissner and colleagues performed a series of histological studies aimed at identifying a magnetite-based magnetoreceptor in the beak. They removed the upper beak skin of pigeons, before sectioning and staining with PB, which labels ferric iron. They reported the identification of six specific patches of PB-positive cells that were located at bilateral locations in the rostral subepidermis of the beak. They showed that these cells had a light blue background stain, contained 10–15 dark blue spherules that were 1 μm in diameter, and a larger oval-shaped structure that was PB negative (Fleissner et al., 2007; Falkenberg et al., 2010). They further claimed that *all* these cells were neurons, were orientated in particular planes, and contained clusters of superparamagnetic magnetite flanked by iron platelets that were aligned in the center of the dendrite. Histological analysis of other avian species led the authors to propose that these cells constituted a conserved magnetic sense system (Falkenberg et al., 2010). In an attempt to replicate the studies of Fleissner and colleagues, we undertook an extremely laborious histological analysis of the upper pigeon beak on a large number of animals (n > 200). Employing the PB stain, we had no difficulty identifying iron-rich cells that mirrored the morphology previously described (Figures 3A–3C); however, careful mapping of these cells across the length of the pigeon beak revealed huge variations in their distribution and number. Some birds had more than 100,000 PB-positive cells, while others had just 200 (Treiber et al., 2012). Staining with three different neuronal markers (neurofilament, TUBB3, and Map1b), and the analysis of more than 2,500 PB positive cells, revealed almost no co-localization (<0.1%) (Figure 3D). These data led us to investigate the subcellular architecture of PB-positive cells in the beak. Contrary to the previous claims, we found that PB-positive cells did not resemble dendrites, were rich in ferritin-like structures, contained membrane bound siderosomes, and had noticeable lamellipodia and filopodia (Figures 3F and 3G). Hypothesizing that these cells were really macrophages, we undertook immunostaining with the antigen-presenting marker MHCII and CD44, revealing high levels of co-localization (≈95%) (Treiber et al., 2012, 2013) (Figure 3E). We think the myth of a 6 loci magnetic sense system in birds can now be put to rest, but what of a trigeminal-mediated magnetic sense? It is possible that the PB method lacks the sensitivity to reveal single domain magnetite in the beak, or perhaps, that a light-based radical pair magnetoreceptor is associated with the trigeminal nerve. It is also worth noting that in other species the trigeminal nerve also projects to the inner ear (Vass et al., 1997), olfactory bulb, and the nasal epithelium (Schaefer et al., 2002). Perhaps, we have just been looking in the wrong place.

## It has to be cryptochrome

The second dominant theory in the field of magnetoreception ventures into the realms of quantum physics. It reasons that photosensitive molecules, when activated by light, produce radical pairs. These radical pairs can exist either in a singlet or triplet state that are interconvertible (Ritz et al., 2000; Mouritsen and Hore, 2012). It is conceivable that the Earth’s magnetic field could alter the ratio of these states, thereby influencing downstream signaling cascades (Figure 2D). Klaus Schulten, the first advocate of this hypothesis, proposed that such a mechanism might rely on light-sensitive proteins—the cryptochromes (Ritz et al., 2000; Schulten K Weller, 1978). Cryptochromes are signaling molecules found in plants, insects, birds, and mammals that function as important regulators of the circadian clock (Cashmore et al., 1999). Some, but not all, cryptochromes bind the flavin co-factor FAD and therefore they can also function as UV/blue light photosensors (Baik et al., 2017). Their candidacy as the molecular mediators of the magnetic sense has been advanced for a number of reasons. First, Wiltschko and colleagues showed that both adult and young migrating birds orient well when exposed to blue (443 nm) or green light (565 nm), but fail to do so under red light (630 nm) (Wiltschko et al., 1993; Wiltschko and Wiltschko, 1995). Second, broadband radio frequency fields which influence the spin states of electrons have consistently been shown to perturb magnetic orientation in behavioral assays (Engels et al., 2014; Ritz et al., 2004). Third, a number of genetic studies in *Drosophila* and Monarch butterflies have shown that magnetoreception requires the presence of light-sensitive cryptochromes (Gegear et al., 2008, 2010; Fedele et al., 2014; Yoshii et al., 2009; Wan et al., 2021). Fourth, there is evidence that the avian cryptochrome CRY4 undergoes structural changes when exposed to blue/green light, which is dependent on a string of aromatic tryptophan residues and the binding of the flavin co-factor FAD (Watari et al., 2012; Niessner et al., 2013). Fifth, recent studies have shown that CRY4 has the capacity to form long-lived radical pairs which are susceptible to magnetic fields (30 mT) (Hochstoeger et al., 2020; Xu et al., 2021). Consequently, CRY4 is rightly considered to be the best candidate magnetoreceptor in birds (Hore and Mouritsen, 2016). There are, however, a number of inconvenient facts that cannot be ignored. There is a notable inconsistency between the absorption spectra of the fully oxidized FAD (320–500 nm) (Evans et al., 2013) which is necessary for radical formation, and the ability of birds to orient in green light (565 nm) (Wiltschko and Wiltschko, 2001). Moreover, all the avian cryptochromes are broadly expressed, including CRY4, which has been found in all retinal cell types. Given this lack of specificity, it is not immediately clear how the animal would distinguish between a visual and magnetic stimulus, or which cells would function as the primary sensors. Notably, in the case of mammals, the current evidence indicates that the two cryptochromes present (CRY1 and CRY2) do not bind FAD, and therefore lack the attributes to function as a magnetoreceptor (Kutta et al., 2017). Furthermore, there is an issue with thermal noise. The Earth’s magnetic field is very small (50 μT), and the environment of any cell is a chaotic one. Simultaneously, organelles are being translocated, genetic material is being transcribed, proteins are being degraded, and a myriad of biochemical reactions are being catalyzed, all at a temperature of 42°C. The energy resulting from this thermal background is more than a million times stronger than the effect of a 50 μT field on the spin state of electrons, attributable to Zeeman splitting (Johnsen et al., 2020). How a cryptochrome molecule would decipher such a small signal when surrounded by so much noise is not clear. Until these issues are addressed, the jury must refrain from delivering its verdict, and the investigation should continue. At present, CRY4 is the only credible suspect, but it is conceivable that another will emerge whose photonic fingerprint better fits the profile.

## MagR is a protein biocompass

In 2016, headlines declared that a magnetic protein biocompass had been discovered, that might serve as a “universal mechanism for animal magnetoreception” (Cyranoski, 2016; Sample, 2016). Amalgamating elements of the light-dependent hypothesis and an iron-based magnetoreceptor, Xie and colleagues employed *in silico* and biochemical methods to identify iron-binding proteins that interact with CRY4 (Qin et al., 2015). They claimed that the iron-sulfur chaperone ISCA1, which they renamed MagR, forms a rod-like polymer and is ferrimagnetic at room temperature. In further support of their assertion that ISCA1/CRY4 forms the molecular basis of the magnetic sensor, they claim that crystals of the complex rotate synchronously with an external magnetic field and that pigeon CRY4 and ISCA1 co-localize in all major cell types in the retina. Subsequent papers have reported the cloning of ISCA1 and CRY4 from medaka and zebrafish arguing that the discovery of the ISCA1/MagR magnetosensory system is a huge step forward in understanding the molecular mechanisms of the magnetic sense (Wang et al., 2017; Zhou et al., 2016). It has been further claimed that ectopic expression of pigeon ISCA1 can be employed for non-invasive magnetogenetic stimulation *in vitro* and *in vivo* (Long et al., 2015). Regrettably, ISCA1 is not tenable as a natural or artificial magnetoreceptor. Firstly, ISCA1 is a chaperone that binds just a few iron atoms and is found in both prokaryotic and eukaryotic organisms (Vinella et al., 2009; Sheftel et al., 2012; Lu et al., 2010). Not surprisingly, this ancient protein is ubiquitously expressed in all cells in vertebrates and therefore lacks the spatially restricted expression pattern that is characteristic of sensory receptors (Hochstoeger et al., 2016). Secondly, and most critically, the ISCA1/CRY4 complex cannot be ferrimagnetic. Ferrimagnetism and ferromagnetism are associated with exchange interactions, which align electron spins resulting in a net magnetic moment (Spaldin, 2010). In the case of an average magnetotactic bacteria, 17 magnetosomes, each with more than 1 million iron atoms (Fe_3_O_4_), generate a moment of 4.2 × 10^−16^ Am^2^/nm^3^ (approximately 2.1 × 10^−20^ J in an Earth strength field) (Nadkarni et al., 2013). In contrast, the ISCA1/CRY4 complex proposed by Xie and colleagues has a total of just 40 iron atoms that are molecularly disparate. The consequence is that the spins of these iron atoms will not be coupled at room temperature (Winklhofer, 2016), and even if they were the energy of the magnetic moment (1 × 10^−25^ J) still fails to exceed thermal background (4 × 10^−21^J) (Meister, 2016). Unsurprisingly, subsequent well-controlled attempts to use ISCA1 as a magnetic actuator have failed (Pang et al., 2017; Wang et al., 2020). The most likely explanation for the magnetic properties of the protein “crystals” reported by Xie and colleagues is contamination from the laboratory environment—a persistent problem that has trapped many (Edelman et al., 2015; Kobayashi et al., 1995; Eder et al., 2012). In addition, it should be noted that the experiments performed by Xie and colleagues employed a truncated version of pigeon CRY4, that lacks the critical C-terminal tail (Hochstoeger et al., 2016). Furthermore, there is no evidence that the ISCA1/CRY4 complex actually exists *in vivo*. We recently generated two pigeon CRY4 monoclonal antibodies, which we employed to identify CRY4 interactors in the retina, liver, and cerebellum. Immunoprecipitation experiments identified a number of known cryptochrome partners (e.g timeless), but not once did we observe any interaction between CRY4 and ISCA1 (Hochstoeger et al., 2020). The ISCA1 magnetoreceptor is an example of a modern myth, and exemplifies why it is imperative to critically analyze experimental data and consider alternative explanations.

## The electromagnetic induction hypothesis is dead

Electromagnetic induction, a phenomenon first described by Michael Faraday, reflects the intimate relationship between electricity and magnetism (Farraday, 1838). Put simply, a changing magnetic field will induce a voltage in a conductor. This voltage is a result of the Lorentz force, which reflects the effect of magnetic fields on charged particles. If a circuit exists, a current will result (Figure 2E). Conceptually, this principle may underlie a magnetic sensor as it translates magnetic stimuli directly into electrical information. This idea has been primarily considered with respect to aquatic animals with an acute electric sense (e.g. sharks and rays) (Lohmann and Johnsen, 2000). It has been postulated that such animals could measure voltage changes due to their movement through a fixed magnetic field. In this model, the inner ear ampullary canals or the long jelly-filled tubes of the ampullae of Lorenzini serve as conductors which move through the magnetic field inducing a current that is detected by sensory neurons, with the surrounding sea water completing the circuit. Might such a mechanism also underlie the magnetic sense in terrestrial animals? This idea has generally been dismissed because the surrounding environment of a bird, air, is not a conductive medium (Johnsen and Lohmannn, 2005). However, it is conceivable that an internal fluid-filled circuit, such as the semicircular canals in the inner ear, could serve as an electromagnetic receptor (Figure 2F). Viguer speculated that within the endolymph of these canals, currents could be induced whose strength would vary depending on their orientation with respect to the inclination, declination, and intensity of the Earth's magnetic field (Viguier, 1882). This concept was further advanced by Jungerman and Rosenblum who proposed that sensory hair cells, located in the ampulla, could serve as voltage sensors, detecting a difference in potential within the semicircular canals (Jungerman and Rosenblum, 1980). For this mechanism to be tenable, it would require an exquisitely sensitive voltage detector. Indeed, it was recently shown that a splice-isoform of the voltage-gated calcium channel Ca_v_1.3, which mediates electroreception in sharks and skates, is expressed in the pigeon auditory and vestibular system (Nimpf et al., 2019). This isoform is characterized by a decreased threshold of activation making it susceptible to minute voltage changes (Bellono et al., 2017, 2018). Furthermore, experimental and theoretical modeling demonstrated that natural head movement can induce electric fields within the pigeon semicircular canals of sufficient strength (7.9–9.6 nV/cm) to be detectable by known electroreceptive systems (5 nV/cm) (Kalmijn, 1982). While the direct conversion of a magnetic stimulus into a physiologically relevant electrical signal might be an attractive hypothesis, there is currently little hard evidence to support it (Winklhofer, 2019). Two groups have reported magnetically induced neuronal activation in the medial vestibular nuclei (VeM) in pigeons in the dark, but it is possible that this merely reflects multimodal sensory integration within the brain stem (Wu and Dickman, 2011; (Nimpf et al., 2019) ). Moreover, the theory fails to provide a compelling explanation for the light-dependent magnetic behavior observed in migratory birds and the reduced activation threshold of Ca_v_1.3 might simply be a means to tune avian hair cells to auditory and vestibular stimuli. Nevertheless, it might be a little premature to dismiss electromagnetic induction at this stage (Wu and Dickman, 2011, 2012).

## Concluding remarks

This review has highlighted a number of scientific myths that have emerged over the years; some have been resolved, others remain contentious. As a community, our challenge is to ensure that facts reign over fantasy. It is imperative that experiments (whether they be behavioral, histological, or neurophysiological), incorporate the necessary controls, a sufficient number of animals, and that the data are quantitated employing strict blinding procedures. The interpretation of these data should be critical, considering alternative explanations. Experiments should be replicated independently by different labs and any proposed mechanism should be considered in light of the physical principles that underlie our universe. Magnetoreceptors that defy them should be questioned (Meister, 2016). Finally, we think it is important that investigators keep an open mind. At conferences, cryptochrome has often faced magnetite on the scientific battlefield, with views becoming more deeply entrenched. Yet, if we are to unravel the mystery of magnetosensation creativity, intellectual flexibility and implementation of modern neurophysiological methods will be indispensable (Malkemper et al., 2020). After all, biology has defied expectation on more than one occasion.
